# Notes on the genus *Chionolaena* in Colombia with a new species *Chionolaena barclayae* (Asteraceae, Gnaphalieae)

**DOI:** 10.3897/phytokeys.46.8976

**Published:** 2015-02-05

**Authors:** Harold Robinson

**Affiliations:** 1 Department of Botany, MRC 166, National Museum of Natural History, P.O. Box 37012, Smithsonian Instititon, Washington DC., 20013-7012

**Keywords:** *Chionolaena*, Colombia, Sierra Nevada de Santa Marta, new species

## Abstract

A new species and a new record for *Chionolaena* are recorded from Sierra Nevada de Santa Marta, Colombia adding to the two species of the genus already known from that mountain complex.

## Introduction

Specimens that had been sent to José Cuatrecasas over the years include many that have remained unidentified and were put aside for later work. One set reported here contained members of the tribe Gnaphalieae from the Sierra Nevada de Santa Marta, in northern Colombia, a still inadequately explored mountain area adjacent to but separate from the Andes. When specimens were first put aside, it was not certain to what genus they belonged. *Pseudoligandra* Dillon & Sagást. was suspected but *Chionolaena* DC. and *Gnaphaliothamnus* Kirpiczn. were possibilities. Recent publications by Freire (1993) and Nesom (2001) have totally resolved that problem by reducing all three genera to synonymy under the name *Chionolaena*. Thus, the position of the Santa Marta material is resolved, and is reinforced by some new observations.

## Results and discussion

Freire (1993) cited a number of features that were characteristic of *Chionolaena*, recurved margins of the leaves, involucral bracts with an undivided stereome, and with spreading, pale, usually white tips on the inner bracts; heads with numerous female florets with reddish filiform corollas, the few usually bisexual florets in the centers of the heads, the connate bases of the pappus bristles and the inflated tips of the pappus bristles. Regarding the connate bases of the pappus bristles, I would offer confirmation, but connation is so short that it is easily missed. Still it is present, even in the type species of *Gnaphaliothamnus* in which Freire believed it was lacking. Nesom (2001) pointed out that the tips of the pappus bristles were not always inflated, but it should be noted that the apical cells are always blunt at the tips.

In a limited study based on hairs pulled from the leaves of numerous species, a possible unifying character has been observed. The hairs were only pulled, not dissected from the leaves, therefore the structure of the bases cannot be stated with certainty, but one feature was consistent as observed. Each hair from various *Chionolaena* species had a prominent swollen ring near the base at the point of a septation, and remnants of only one thin-walled cell was seen below the septation. This hair type is illustrated by Freire (1993) and the base is shown complete with one thin-walled cell, and one short thick-walled cell basal to that. Only one species of *Pseudognaphalium* Kirpiczn. *Pseudognaphalium meridanum* (Aristeg.) Anderb., was tested in comparison, and the ring at the septation was much less prominent, and there were 2-3 longer thin-walled basal cells. Further studies on this are suggested.

In the monograph by Freire (1993), two species of *Chionolaena* were already recognized from The Sierra Nevada de Santa Marta, *Chionolaena chrysocoma* (Wedd.) Freire and *Chionolaena colombiana* S.F. Blake. The present study shows that two additional species of the genus occur on the mountain.

The new records of *Chionolaena* from the Sierra Nevada de Santa Marta are as follows:

***Chionolaena Salicifolia* (Bertol.) Nesom, SIDA 19(4): 850. 2001.**

*Helichrysum salicifolium* Bertol. Nov. Comm. Acad. Sci. Bonon. 4: 433. 1840.

*Gnaphalium seemannii* Sch.Bip. in Seemann, Not, Voy. Herald 309. 1856.

*Gnaphalium rhodanthum* Sch. Bip. in Seemann, Bot. Voy. Herald 310. 1856.

*Chionolaena corymbosa* Hemsley, Diagn. Pl. Nov. 2: 32. 1879

*Gnaphaliothamnus rhodanthus* (Sch.Bip.) Kirpiczn, Trudy Bot. Inst. Akad. Nauk SSSR, ser 1, Fl. Sist. Vyss, Rast. 9: 33. 1950 (type of *Gnaphaliothamnus*).

*Gnaphaliothamnus salicifolius* (Bertol.) Nesom, Phytologia 68: 378. 1990.

*Chionolaena seemannii* (Sch.Bip.) Freire, Ann. Missouri Bot. Gard. 80: 452. 1993.

The synonymy follows Nesom (2001) rearranged according to order of publication. Specimens from Santa Marta are as follows:

**Colombia.** Depto. Magdalena, Sierra Nevada de Santa Marta, on trail above San Pedro de la Sierra. Paramo 3900 m, small terrestrial herb, 29 Dec, 1974, ; *R.J. Robins & E.J. Kirby 618A* (Fielding Herbarium, OXF). *R.J. Robins 618B* (OXF, frag US)

The specimens differ from Mexican and Central American material examined of the species only in having glabrous achenes.

### Chionolaena barclayae

Taxon classificationPlantaeAsteralesAsteraceae

H. Rob. sp. nov.

urn:lsid:ipni.org:names:77145081-1

#### Type.

**Colombia.** Depto. Magdalena. Sierra Nevada de Santa Marta; alrededores de cabeceras de Río Ancho; Páramo de Macotama, above and west of second lake; above valley of Río Ancho, Sta. 15, alt. 4900-5000 m. On high outcrops of bedrock. Shrub, erect stems branching from woody base, to 20 cm tall, entire stem and leaves and involucre gray, hairy, heads yellowish, dry. 17 Feb. 1959. *Harriet G. Barclay & Pedro Juajibioy 7072* (holotype US, isotype COL).

#### Description.

Small shrubs to 20 cm tall. Stems branched distally. Leaves alternate, imbricated, appressed, broadly inserted and membraneous at base; blade oblong. 4 mm long by ca. 1.5 mm wide, coriaceous with narrowly recurved margins, dark green, covered with pale hairs on both surfaces, longer and more yellowish abaxially, abaxial pubescence dense and giving abaxial leaf surface rounded appearance, completely obscuring leaf margins, apex blunt. Infloresence of mostly 1–3 heads at tips of unattenuated branches; heads hemispheric, ca. 7 mm high, to 4 mm wide; pale- tipped involucral bracts ca. 15, narrowly lanceolate, 4–5 mm long. ca. 0.8 mm wide. with distal ca. 1.5 mm usually reflexed and whitish inside, pale pink outside; peripheral functionally female florets ca. 20 or more; corollas reddish, filiform, ca. 3.5 mm long, with pair of minute lobes and small biseriate glands distally; style base enlarged, distal branches filiform, scarcely roughened; achenes ca. 1 mm long; glabrous; pappus bristles ca. 28–30, ca. 4 mm long, bases connate in basal row of cells, tips not or scarcely broadened, apical cells with blunt tips; bisexual florets 3–6; corollas reddish, narrowly funnelform, ca. 3.5 mm long, distally with 5 lanceolate lobes ca. 0.5 mm long; anther thecae ca. 0.7 mm long, with long basal tails, apical appendage oblong-lanceolate, ca. 0.5 mm long, glabrous; style base enlarged, distal branches narrowly lanceolate. acute at tip, papillose on sides and apex; achene ca. 1 mm long, glabrous; pappus bristles ca. 28, connate in basal row of cells, distally broadened with bulging cells.

The species is known only from the single collection by Harriet Barkley and Juajibioy. The species is evidently closest to *Chionolaena chrysocoma*, also from Santa Marta, which also has appressed imbricated leaves. The new species has much broader leaves with dense pubescence abaxially that makes the abaxial surface seem rounded and completely hides the recurved leaf margins. Although the collector stated the heads were yellowish, the bracts and corollas seem reddish or pink. The differentiated tips of the involucral bracts seem less white than in other members of the genus.

The habit of the new species somewhat resembles that of *Chionolaena costaricensis* (Nesom) Nesom, but the Costa Rican species has much less densely pubescent leaves that are most often spreading rather than appressed to the stem.

**Figure 1.**
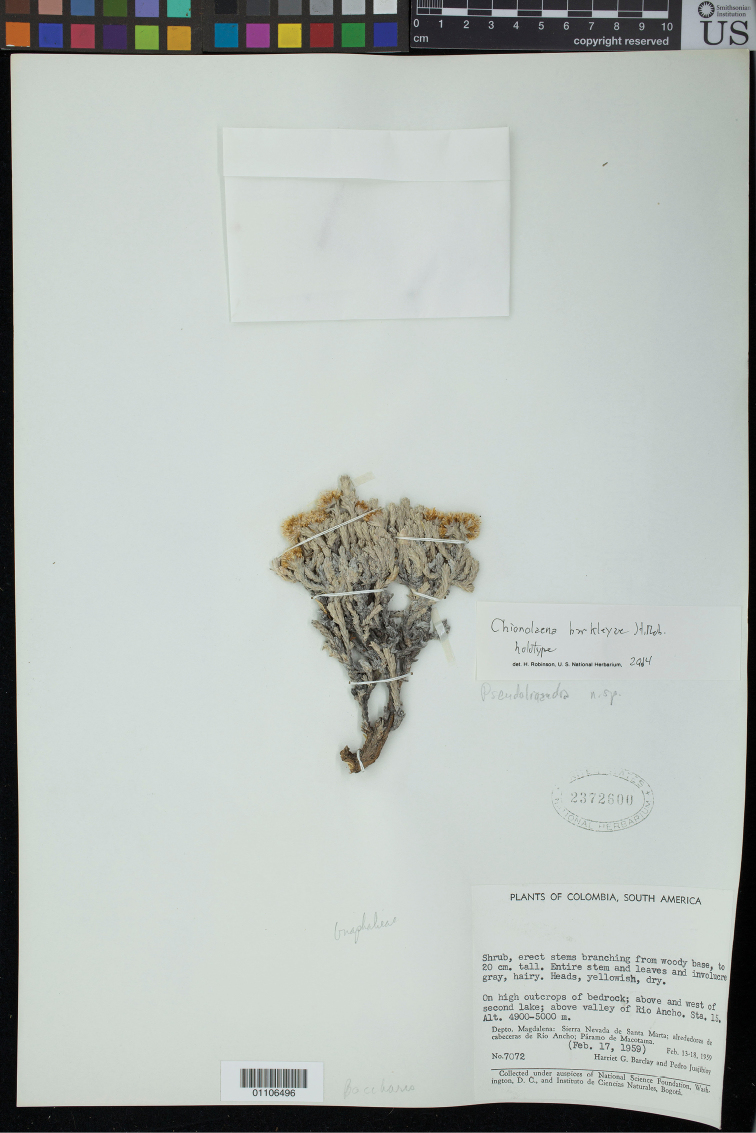
Holotype of *Chionolaena barclayae* H. Rob., *Barxlay & Juajibioy 7072* (US).

## Supplementary Material

XML Treatment for Chionolaena barclayae
